# P-2055. Modelling the Relative Vaccine Effectiveness of ARCT-154 versus BNT162b2 in Younger and Older Adults

**DOI:** 10.1093/ofid/ofae631.2211

**Published:** 2025-01-29

**Authors:** Van Nguyen, Pascal Crepey, Jean Marie Pivette, Ettan Settembre, Sankarasubramanian Rajaran, John Youhanna, Aimee Ferraro, Cheng Chang, Josephine Van Boxmeer, Joaquin F Mould-Quevedo

**Affiliations:** VHN Consulting, Montreal, Quebec, Canada; University of Rennes, Rennes, Bretagne, France; VHN Consulting, Montreal, Quebec, Canada; CSL Seqirus USA, Boston, Massachusetts; CSL Seqirus UK, Maidenhead, England, United Kingdom; CSL Seqirus USA, Boston, Massachusetts; CSL Seqirus USA, Boston, Massachusetts; CSL Seqirus USA, Boston, Massachusetts; CSL Seqirus USA, Boston, Massachusetts; CSL Seqirus Inc., Summit, New Jersey

## Abstract

**Background:**

While vaccines have proved a key tool in protection from COVID-19, the duration of immunity induced by first-generation mRNA vaccines is limited. Self-amplifying mRNA (SA-mRNA) vaccines have clinically shown to both elicit higher antibody responses and increase the duration of response. In this analysis, we evaluate the relative vaccine effectiveness (rVE) over time of a COVID-19 SA-mRNA vaccine (ARCT-154) versus BNT162b2 against the original Wuhan-Hu-1 and Omicron BA.4/BA.5 strains in adults stratified by age group (< 60 years and ≥60 years), using a correlate of protection derived from immunogenicity data.

**Methods:**

We used geometric mean titer data collected on Days 29, 91, and 181 post-vaccination from a phase 3 clinical trial comparing the immune response of ARCT-154 and BNT162b2 against Wuhan-Hu-1 and Omicron BA.4/5 in healthy 18–77-year-olds (Japan Registry for Clinical Trials identifier: jRCT2071220080) to estimate a correlate of protection against symptomatic and severe COVID-19. rVE of ARCT-154 versus BNT162b2 was then evaluated for each time point against both endpoints in individuals < 60 years and ≥60 years of age.

**Results:**

rVE against symptomatic infection and severe illness increased over time against both Wuhan-Hu-1 and Omicron BA.4/BA.5. In adults < 60 years, rVE against symptomatic infection ranged from 2.57% on Day 29 to 9.19% on Day 181 against Wuhan-Hu-1 and from 4.24% to 26.83% against Omicron BA.4/5. In adults ≥60 years, rVE ranged from 2.77% to 10.99% and 4.58% to 48.02% against the two strains, respectively. rVE against severe illness ranged from 0.30% to 4.65% against Wuhan-Hu-1 and 0.63% to 15.84% against Omicron BA.4/5 in adults < 60 years and from 0.34% to 5.61% and 0.74% to 27.96%, respectively, in adults ≥60 years.

**Conclusion:**

ARCT-154 showed longer-lasting protection than BNT162b2 against symptomatic and severe COVID-19, with greatest benefits in adults ≥60 years of age against Omicron BA.4/BA.5.

**Disclosures:**

Van Nguyen, PhD, CSL Seqirus USA: Advisor/Consultant Pascal Crepey, n/a, CSL Seqirus USA: Advisor/Consultant Jean Marie Pivette, n/a, CSL Seqirus USA: Advisor/Consultant Ettan Settembre, n/a, CSL Seqirus USA: Stocks/Bonds (Private Company) Sankarasubramanian Rajaran, n/a, CSL Seqirus UK: Stocks/Bonds (Private Company) John Youhanna, n/a, CSL Seqirus USA: Stocks/Bonds (Private Company) Aimee Ferraro, n/a, CSL Seqirus USA: Stocks/Bonds (Private Company) Cheng Chang, n/a, CSL Seqirus USA: Stocks/Bonds (Private Company) Josephine Van Boxmeer, n/a, CSL Seqirus Amsterdam: Stocks/Bonds (Private Company) Joaquin F. Mould-Quevedo, PhD, CSL Seqirus USA: Stocks/Bonds (Private Company)

